# Neutropenia in patients under treatment with clozapine and COVID-19 infection

**DOI:** 10.1192/j.eurpsy.2021.1796

**Published:** 2021-08-13

**Authors:** S. Bonaccorso, A. Ricciardi, S. Ouabbou, C. Theleritis, A. Ross-Michaelides, A. Metastasio, N. Stewart, M. Mohammed, F. Schifano

**Affiliations:** 1 Highgate Mental Health Centre, Camden & Islington NHS Foundation Trust, London, United Kingdom; 2 1st Psychiatry Dept, National and Kapodistrian University of Athens, Athens, Greece; 3 Psychiatry And Psychopharmacology, University of Hertfordshire, Hertfordshire, United Kingdom

**Keywords:** clozapine, COVID-19, neutropenia, schizophrénia

## Abstract

**Introduction:**

Clozapine is among the most effective antipsychotics used for treatment resistant schizophrenia. Adverse reactions to clozapine include neutropenia. Case series report that clozapine-treated patients with COVID-19 have no documented neutropenia.

**Objectives:**

We sought to investigate the potential adverse effect of coronavirus disease (COVID-19) in patients taking clozapine.

**Methods:**

We retrospectively inspected data of 13 consecutive patients on clozapine, admitted to Highgate Mental Health Centre -Camden & Islington NHS Foundation Trust between March and June 2020. Selection was based on their COVID-19 symptoms presentation and/or COVID-19 positive test. We used a linear regression model with COVID status as independent variable and absolute neutrophil count (ANC) as dependent variable to inform about a correlation between COVID-19 status and neutrophil count. STATA was used for statistics.

**Results:**

We collected data on thirteen patients of which nine were male. The median age was of 41.97 years; six subjects were Black, three were Asian and four were White Caucasian. Ten subjects tested positive to COVID-19 and 3 were suspected cases -these latter were excluded from stastical analysis. During COVID-19 infection, neutrophils count (ANC) dropped significantly to 4.215 from a baseline value of 5.337. The beta values of 0.83 shows that ANC declined significantly during COVID-19 infection (p =<.0001, R^2^ = 95%). In three of thirteen patients, ANC drop was significant and changed the patients’ monitoring status from green to amber and required frequent blood tests.

**Conclusions:**

Clinicians should bear in mind that a significant drop in neutrophils count may occur in COVID-19 -infected patients taking clozapine.

**Disclosure:**

No significant relationships.

